# The Pivotal Role of the Placenta in Normal and Pathological Pregnancies: A Focus on Preeclampsia, Fetal Growth Restriction, and Maternal Chronic Venous Disease

**DOI:** 10.3390/cells11030568

**Published:** 2022-02-06

**Authors:** Miguel A. Ortega, Oscar Fraile-Martínez, Cielo García-Montero, Miguel A. Sáez, Miguel Angel Álvarez-Mon, Diego Torres-Carranza, Melchor Álvarez-Mon, Julia Bujan, Natalio García-Honduvilla, Coral Bravo, Luis G. Guijarro, Juan A. De León-Luis

**Affiliations:** 1 Department of Medicine and Medical Specialties, Faculty of Medicine and Health Sciences, University of Alcalá, 28801 Alcalá de Henares, Madrid, Spain; oscarfra.7@hotmail.com (O.F.-M.); cielo.gmontero@gmail.com (C.G.-M.); msaega1@oc.mde.es (M.A.S.); maalvarezdemon@icloud.com (M.A.Á.-M.); diegotc90@gmail.com (D.T.-C.); mademons@gmail.com (M.Á.-M.); mjulia.bujan@uah.es (J.B.); natalio.garcia@uah.es (N.G.-H.); 2 Ramón y Cajal Institute of Healthcare Research (IRYCIS), 28034 Madrid, Spain; luis.gonzalez@uah.es; 3 Cancer Registry and Pathology Department, Hospital Universitario Principe de Asturias, 28801 Alcalá de Henares, Madrid, Spain; 4 Pathological Anatomy Service, Central University Hospital of Defence-UAH, 28047 Madrid, Spain; 5 Immune System Diseases-Rheumatology and Oncology Service, University Hospital Príncipe de Asturias, CIBEREHD, 28801 Alcalá de Henares, Madrid, Spain; 6 Department of Public and Maternal and Child Health, School of Medicine, Complutense University of Madrid, 28040 Madrid, Spain; cbravoarribas@gmail.com (C.B.); jaleon@ucm.es (J.A.D.L.-L.); 7 Department of Obstetrics and Gynecology, University Hospital Gregorio Marañón, 28009 Madrid, Spain; 8 Health Research Institute Gregorio Marañón, 28009 Madrid, Spain; 9 Unit of Biochemistry and Molecular Biology (CIBEREHD), Department of System Biology, University of Alcalá, 28801 Alcalá de Henares, Madrid, Spain

**Keywords:** placenta, preeclampsia, fetal growth restriction, maternal chronic venous disease (CVeD)

## Abstract

The placenta is a central structure in pregnancy and has pleiotropic functions. This organ grows incredibly rapidly during this period, acting as a mastermind behind different fetal and maternal processes. The relevance of the placenta extends far beyond the pregnancy, being crucial for fetal programming before birth. Having integrative knowledge of this maternofetal structure helps significantly in understanding the development of pregnancy either in a proper or pathophysiological context. Thus, the aim of this review is to summarize the main features of the placenta, with a special focus on its early development, cytoarchitecture, immunology, and functions in non-pathological conditions. In contraposition, the role of the placenta is examined in preeclampsia, a worrisome hypertensive disorder of pregnancy, in order to describe the pathophysiological implications of the placenta in this disease. Likewise, dysfunction of the placenta in fetal growth restriction, a major consequence of preeclampsia, is also discussed, emphasizing the potential clinical strategies derived. Finally, the emerging role of the placenta in maternal chronic venous disease either as a causative agent or as a consequence of the disease is equally treated.

## 1. Introduction

The placenta is an intricate and vital organ during pregnancy, coordinating a wide variety of functions in this period. Synchronically with the fetus, the placenta is an organ that experiences incredible transformation and growth from its early development to the end of pregnancy [1]. The placenta could be considered the mastermind behind maternal physiology, orchestrating an entire organism to create a proper milieu for fetal development [2]. When this structure does not work properly, it could lead to the onset of different pregnancy complications, with serious consequences for maternofetal well-being [3]. Despite this organ being only with us during gestation, its relevance extends far beyond this period. Indeed, the fetus perceives the placenta as a reflection of the outer environment, and the different signals received through this organ may have important consequences in a newborn and even in adulthood [4]. Thus, a study of the placenta prominently progresses our understanding of human health and disease [5]. On the other hand, due to the transitory and dynamic nature of the placenta, little information could be obtained from the placenta in real time, hampering examinations of this organ. To shed light on this issue, some projects, such as The Human Placenta Project, aim to deeply explain the development, structure, and function of this complex organ [6]; however, the road to gaining further insights into the complex and critical role of the placenta during pregnancy is still very long.

In this context, the purpose of this review is to collect updated knowledge about the placenta in uncomplicated pregnancies, with the aim to understand the steps of placentation and placental growth, the cytoarchitecture of this structure, its immunology, its multiple activities, and the modulatory role of this organ in fetal development and maternal physiology, hence creating a global picture of this organ. In the second part, we focus on the role of the placenta in the pathophysiology of two hypertensive disorders: preeclampsia (PE) and chronic venous disease (CVeD). We also analyze the relevance of this organ with fetal growth restriction.

## 2. Placental Development, Cytoarchitecture, and Immunology

### 2.1. Early Development of the Placenta 

The placenta is an organ that develops during pregnancy in a gradual and poorly understood process. Currently, after fecundation and successive cell divisions, the embryo undergoes complex interactions with a receptive uterus in the form of blastocysts. Consequently, if the implantation process is successful, the embryo attaches to the endometrium, invading the epithelium and maternal circulation, initiating the process of placentation [7]. During the implantation process, the blastocyst differentiates into an inner cell mass (embryo) and trophectoderm (placenta). The trophectoderm houses trophoblasts—the main drivers of the placentation process and different placental functions [8]. Simultaneously, the stromal cells in the maternal endometrium surrounding the implanting embryo develop a plethora of changes in a process designated as decidualization, which is an imperative prerequisite for implantation success [9]. Trophoblasts are semi-allogeneic cells, as they are derived from the embryo. In this sense, a possible rejection of these cells by the maternal immune system would likely be fatal. However, trophoblasts exert multiple immunoregulatory actions, leading to a maternofetal tolerance response, hence ensuring placentation and pregnancy success [10]. 

Just after implantation, a syncytial fusion of mononucleated trophoblasts forms the oligonucleated syncytiotrophoblasts (STBs). The remaining mononucleated trophoblasts are referred to as cytotrophoblasts (CTBs) [11]. The sequence of processes involved in the formation of the placenta is as follows: (1) In the prelacunar stage, the fusion of mononucleated cells leads to the formation of the first STBs or primary syncytium. This syncytium is the outer layer of the placenta and is in direct contact with the maternal blood, and as described later, these cells represent a major structural and functional unit of the placenta. (2) In the lacunar stage, fluid-filled spaces—named lacunae—appear within the central mass of the primary syncytium. STBs surrounding the lacunae are named trabecula. This phase occurs from day 8 to day 13 after conception. The system of trabecula and lacunae is coated with two layers free of lacunae: the basal layer, facing the endometrium, known as the cytotrophoblast shell, and a superficial layer in contact with the blastocyte, called the primary chorionic plate [12]. In these lacunae, some evidence of maternal circulation can be found, coming from the erosion of spiral arterioles and small veins from the endometrium after the invasion of this structure [13]. However, maternal circulation in the placenta is not well-established until the end of the first trimester, when hypoxia is essential for the growth and physiological development of the embryo, and the placenta is beneficial [14]. On day 12 after conception, a bilayer structure composed of the CTBs, and extra-embryonic mesodermal cells (somatopleure) is formed. This structure is the chorion, and it represents the fetal part of the placenta [15]. CTBs coming from the chorionic plate invade the syncytial mass of the trabecula, and on day 15, they reach the maternal side of the placenta, leading to their transformation in a special subtype of extravillous trophoblasts (EVTs), named endovascular trophoblasts (eEVTs) and interstitial trophoblasts (iEVTs). iEVTs remain in the endometrial decidua, whereas eEVTs start remodeling the spiral arteries, concluding with the replacement of the endothelium and the smooth muscle cells from the tunica media by trophoblasts. This leads to a set of changes in the properties of these vessels, including loss of elasticity or of vasomotor control [16]. (3) The villous stage starts between days 12 and 18 after conception. The trophoblastic trabecula starts to proliferate, forming protrusions into the maternal blood surrounding the trabecula (primary villi), which is composed of a CTB core with an outer layer of STBs [17]. Then, the extra-embryonic mesodermal cells of the chorionic plate invade the trabecula, although they stop in the distal part exclusively filled with CTBs. These are referred to as the trophoblastic cell column, being a source of EVTs [11]. In addition, the extra-embryonic mesodermal cells invade the primary villi, giving them a mesenchymal core and transforming them into secondary villi. On days 18–20, fetal capillaries appear in the core of the secondary villi, leading to the development of tertiary villi. During the first trimester of pregnancy, a system of villous trees is formed by further proliferation and branching, and the lacunae become the intervillous space. Then, the blueprint of the placenta is established [18].

### 2.2. Placental Anatomy and Cytoarchitecture

During the second and third trimesters of pregnancy, the placenta is an organ with rapid development and multiple changes occurring in this structure as well as in the fetus. During weeks 10–12 after fecundation, the average weight of the placenta is about 51 g, whereas the delivered or mature placenta is essentially a discoid organ with a weight of 500–600 g, a diameter of 22 cm, and a thickness of 2–4 cm. These values may vary under abnormal or pathological pregnancies [19,20]. The human placenta is composed of a fetal surface or chorionic plate, covered by the amnion, to which the umbilical cord attaches, and a maternal surface or decidual basal plate in contact with the endometrium. Between those plates is the intervillous space, in which a set of fetal villous trees (or chorionic villi) project. Overall, at least five types of villous trees have been described according to their developmental stage, structure, vessel-cell type components, vessel branches, and histologic features. Herein, the main types of villi and their features are summarized, although more detail can be found in specialized literature [21,22,23,24]. (1) Mesenchymal villi: initially, all tertiary villi are of this type. They are where the villi proliferate and perform virtually all of the endocrine activities of the placenta. At term, these villi represent less than 1% of the villous volume, as they differentiate into immature intermediate villi in the first and second trimesters and into stem villi in the third trimester. (2) Immature intermediate villi represent an advanced but immature continuation of mesenchymal villi. They may be considered the growth centers of villous trees, working as the main site of exchange during the first and second trimesters, where terminal villi have not yet differentiated. (3) Stem villi are characterized by a condensed fibrous stroma, large vessels, and microvessels and are responsible for supporting the structures of villous trees, having no impact on the endocrine activity and maternofetal exchange in the placenta. (4) Mature intermediate villi present a higher degree of fetal vascularization, important for maternofetal exchange as well as terminal villi formation. (5) Terminal villi are linked to stem villi by intermediate structures and present a high degree of capillarization and dilated sinusoids, making them a proper location for diffusive exchange. Indeed, terminal villi are critical for the transfer of oxygen/carbon dioxide, electrolytes, and nutrients between the mother and fetus. After delivery, the mature placenta consists of 15 to 28 subunits designated as “cotyledons”, which are perfusion chambers partly or completely separated from others by connective tissue and irrigated by one or more maternal spiral arteries. Each cotyledon contains one or more fetal villous tree(s), a fetal artery, and a vein. What determines the greatest or lowest number of cotyledons remains elusive [25]. Stem villi are the major structural units of cotyledons. Each stem villous branches into 3–5 intermediate villi, which in turn divide into 10 to 12 terminal villi. These terminal villi represent 40% of the total villous volume, and most of them float in the intervillous space, although others are attached to the decidua, favoring structural stability for the placenta [23]. 

Notwithstanding that the cytoarchitectures of villi are slightly different according to type, the following integrators may be distinguished: (A) STBs are the continuous, specialized layer of epithelial cells that are in contact with the maternal blood, orchestrating maternofetal exchange. (B) CTBs are highly proliferative cells that may lead to two trophoblastic phenotypes: (1) the villous phenotype, which leads to the development of multinucleated STBs, and (2) the extravillous phenotype in CTBs that detach from placental villi and could be differentiated into iEVTs, invading the endometrial decidua and eEVTs responsible for remodeling of maternal spiral arteries [8]. (C) Fixed and free connective tissue cells are derived from the differentiation of mesenchymal cells and include fibroblasts, endothelial cells, smooth muscle cells, myofibroblasts, or macrophages that can be found at different proportions in the stroma. In the case of macrophages, they are named Hofbauer cells. Despite them being firstly differentiated from mesenchymal cells (even with prior placental circulation established), the latter recruitment of circulating monocytes also enhances the population of these cells [22]. These cells play key roles in angiogenesis, defense, immunomodulation, and villi remodeling [24]. (D) Fetal vessels comprise capillaries and sinusoids in the terminal villi, surrounded by a basement membrane, arteries and arterioles in the stem, and intermediate villi with tunica media, without elastic laminae, but with a more mature endothelial layer in comparison with venules and veins. Importantly, the lumen of these vessels is controlled by autocrine and paracrine factors, as the placenta lacks any innervation [11]. (E) Fibrinoid consists of two different kinds of extracellular-deposited materials known as fibrin-type fibrinoid and matrix-type fibrinoid. The former is composed of fibrin and maternal blood-clot products regulating the growth of villous trees and adapting the intervillous space to blood flow. The latter is a secretory product of EVTs, containing laminins, collagen IV, and heparan sulfate. These components orchestrate trophoblast invasion by interacting with cell surface integrins [26]. On the whole, the placenta is a dynamic and complex organ, and multiple cells, products, and formed structures are essential for a successful pregnancy. However, another central component, the immune system, is equally important.

### 2.3. Immunology of the Placenta

Pregnancy is notably an immune-mediated process, involving complex interactions between the semi-allogeneic fetal cells and maternal immune cells [10]. The interplay between these cells not only prevents immune rejection but also favors and creates an appropriate environment for pregnancy [27]. The placenta is a site at which the majority of immunomodulatory actions occur. The communication between maternal immune cells and fetal trophoblasts is bidirectional, and they are essentially mediated by direct contact and through the release of a plethora of autocrine, paracrine, and endocrine signals, including cytokines, growth factors, and adhesion molecules expressed on the surface of cells, such as integrins, cadherins, selectins, and immunoglobulins [7]. Through these mechanisms, placental trophoblasts orchestrate the actions of resident decidual cells, also influencing the recruitment of circulating leukocytes to the maternofetal interface [28]. In this section, we summarize the main immunobiology of the placenta. 

Many maternal immune cells inhabit the endometrial decidua, including decidual natural killers (dNKs), macrophages, T cells, and dendritic cells (DCs) [29]. dNKs are a major population of leukocytes at the maternofetal interface (70% of the total) [30]. Through several pathways, dNKs mainly interact with the EVT located in the decidua, being centrally involved in fetal tolerance, EVT invasion, and spiral artery remodeling [31]. Trophoblast release of interleukin-15 (IL-15) seems to promote dNK maturation, and these cells promote decidual remodeling through the production of several cytokines, such as interferon-gamma (IFN-γ), vascular endothelial growth factor (VEGF), tumor necrosis factor–α (TNF-α), IL-8, and the chemokine (C-X-C motif) ligand 10 (CXCL10) [32]. Moreover, dNKs are also major mediators of the immune response against several pathogens such as toxoplasmosis or human cytomegalovirus (HCMV) [33]. Recently, three different subsets of NKs have been identified (dNK1, dNK2, and dNK3) [34]. These different subsets are characterized by expressing distinctive receptors and cytokine profiles and exerting different immunomodulatory actions. Decidual macrophages are the second population most commonly found in the endometrial decidua, representing around 20% of the total [30]. Similar to dNKs, Jiang and Wang [35] identified three subpopulations of macrophages according to the expression of C-C chemokine receptor type 2 (CCR2) and the glycoprotein CD11c, distinguishing between CCR2 negative CD11c low, the most abundant (~80%); CCR2 positive CD11c high (10–15%); and CCR2 negative CD11c high, which was the lowest (~5%). Through a transcriptomic analysis, they suggested that CCR2 positive CD11c high subsets were pro-inflammatory M1-like macrophages in vivo, whereas the remaining CCR2 negative subsets were more likely anti-inflammatory M2 macrophages. Thus, under non-pathological conditions, decidual macrophages are an M2 anti-inflammatory phenotype. The main functions of decidual macrophages are tissue remodeling and repair, debris clearance, angiogenesis, and immune tolerance. These macrophages produce the enzyme indoleamine 2,3-dioxygenase (IDO), which catabolizes tryptophan and hinders inflammatory T cell activation [32]. However, due to infections or an aberrant inflammatory environment, these macrophages may switch to an M1 phenotype, associated with the development of multiple pregnancy complications [36]. T cells represent up to 10 to 15% of the total decidual cells, although its presence progressively increases during the later phase of pregnancy [37]. About 45–75% are CD8 T cells or cytotoxic T lymphocytes (CTLs), and ~30–45% of the cells are CD4 T cells or T helper (Th) cells [38]. CTLs are relevant cells implicated in fetal tolerance while providing an immune defense against pathogens and viral infections [39]. Th cells are prominent regulators of pro-inflammatory/anti-inflammatory status in the maternofetal structures [37]. Effector Th mainly includes three central polarizations: (A) Th1 with pro-inflammatory actions and potentially associated with allograft rejection and pregnancy pathologies; (B) Th2, less harmful for the embryo and inversely associated with Th1 polarization; and (C) Th17, probably involved in acute inflammatory events such as infections [38]. In addition, another crucial type of T CD4+ cells named regulatory T cells (Treg) are crucial for the induction and maintenance of tolerance, especially for implantation and during the first stages of pregnancy [40]. Interestingly, the proportion and populations of each T cell subtype appear to vary during pregnancy. Thus, during the first trimester, Treg and Th1 seem to be the predominant cells, with little implication of both Th17 and Th2, creating a pro-inflammatory but controlled environment that is critical during the early stages [41]. The second trimester of pregnancy is more favorable for the mother and is mainly anti-inflammatory, with predominant Th2 responses. The third trimester and, especially, parturition is again a pro-inflammatory status, aiding the expulsion of the fetus and the placenta [42]. 

**Figure 1:**
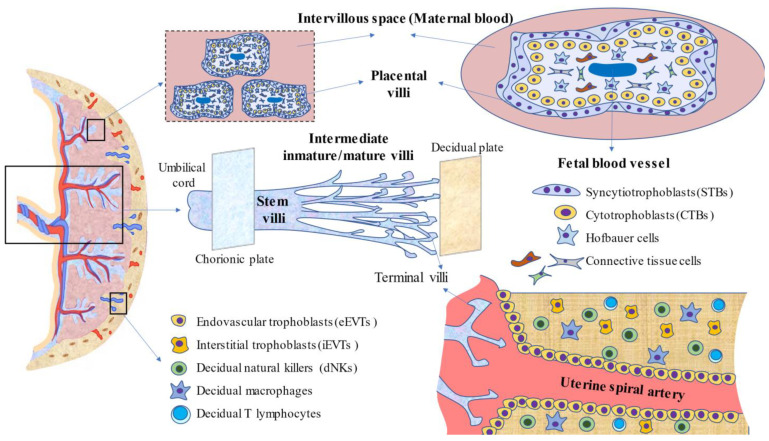
An integrative picture of the components of the placenta. Herein, the main cell types and structures formed are summarized. At the top, placental villi and their cells, as well as the location of the maternal and fetal blood, in the intervillous space and inside the villi, respectively, are represented. In the center of the image, the villous tree, as well as the different types of villi, are represented. The chorionic plate, or fetal surface, is covered by the amnion, where the umbilical cord is attached. The decidual basal plate or maternal surface is in contact with the endometrium. As represented at the bottom, profound remodeling of the uterine spiral arteries is mainly due to the coordinated efforts of a set of cells, mainly extravillous trophoblasts (EVTs) and immune cells, prominently represented by decidual natural killers (dNKs). Having complete knowledge of the placenta in non-pathologic pregnancies is crucial for the study of different pregnancy complications, as the placenta is responsible for a wide variety of functions, as is subsequently discussed.

Globally, the role of immune systems in the placenta, especially the maternal part (decidua basal plate), is crucial for developmental and gestational success. In Figure 1, the main structures and cells present in the human placenta are summarized to create an integrative perspective of this organ. 

### 2.4. Function and Activity of the Placenta during Pregnancy

Despite the relatively short time that the placenta is kept in the women’s body, it should be considered the most important organ in pregnancy. Adequate functioning of this organ is critical for fetal well-being, and substantial alterations of this structure are related to the future development of chronic maladies in the offspring [43]. The essential functions of the placenta that are collected in this section include the following: maternofetal exchange, endocrine activity, barrier and defense activity, and fetal programming. 

#### 2.4.1. Placenta in Maternofetal Exchange

Maternofetal exchange is achieved through a wide variety of mechanisms, with STBs and fetal endothelial cells being two of the major mediators of this process [44]. As mentioned above, maternofetal exchange occurs in successive placental villi, with the terminal villi being those in which this process is conducted in the mature placenta. The mechanisms described in the maternofetal exchange are: (1) bulk flow/solvent drag, which implies that the movement of water and solutes are in favor of the pressure gradient; (2) net solute diffusion, depending on the concentration gradient (e.g., respiratory gases); (3) transport mediated by proteins, where different molecules are implicated in the transport (e.g., nutrients such as glucose, amino acids, ions, or fatty acids); and (4) transcytosis, a combined process of endocytosis and exocytosis (e.g., immunoglobulin G). Two potential mechanisms of transport also occurring in the placenta, such as paracellular transport and the transplacental electrical potential difference, still require much more studies to be understood [45]. Regarding nutrient exchange between the placenta to the fetus, three different mechanisms have been reported: direct transfer from the maternal blood to the fetus, placental intake of nutrients, or placental metabolism of nutrients to alternate substrate forms [46]. Indeed, the placenta is a notable metabolic organ with high oxygen and glucose consumption rates and exerts other reactions related to glucose (glycolysis, gluconeogenesis, and glycogenesis), lipids (lengthening or shortening of fatty acids and triglyceride synthesis), and protein metabolism (protein synthesis and amino acid interconversion) [47]. Inappropriate nutrient supply from the placenta to the fetus is associated with different pregnancy complications [48]. In this sense, previous studies have identified a critical role for the nutrient sensor mammalian target of rapamycin (mTOR) as a link between maternal nutrient availability and fetal growth, thereby representing an interesting marker of fetal well-being [49]. Another critical nutrient, oxygen supply to the fetus, depends on blood oxygen content and flow rate in the uterine and umbilical arteries, as well as the diffusing capacity of the placenta and oxygen use by the placenta, which may occasionally represent a relevant limitation on oxygen availability [50]. Under hypoxic conditions, the placenta may adapt to this change and enhance anaerobic glycolysis in order to ensure an adequate oxygen flow to the fetus. However, this situation may lead to decreased fetal growth as the nutrient supply to the fetus is diminished [51]. The application of various mathematical models is being investigated in order to predict placental exchange efficiency, which may be of great aid as a diagnostic or prognostic tool in terms of mother and fetus health risk [52]. Thus, the fact that the placenta nutrient exchange not only depends on the direct transfer from maternal to fetal blood but also on the metabolic status, morphology, and nutrient utilization of the placenta must be considered. Interestingly, previous studies have found that the placental exchange function may be different across male and female fetuses. Barapatre et al. [53] showed that there was a noteworthy variation in the number of female cell nuclei in STBs in comparison with those detected in males. These changes may be due to the differential environment created by sex chromosomes and may affect the nutrient supply of the placenta, and significant differences may be observed between both sexes [54]. Moreover, some authors extend the function of STBs beyond the villi at the maternofetal interface. This is the case of trophoblast debris, which ranges from multinucleated syncytial nuclear aggregates to subcellular micro and nanovesicles. The former consists of fragments from the STBs with two possible origins: (A) They derive from newly formed villi that start to sprout from existing villi, more prominently during early stages [55]. (B) They come from aged and late apoptotic STBs nuclei, which are packed into apical protrusions, forming what is known as syncytial knots. These structures are released by the STBs in maternal circulation, reaching the lungs where the local macrophages phagocyte this structure, without the activation of any inflammatory response [56]. The micro and nanovesicles (exosomes) are molecules released by the STBs that are crucial for cell-to-cell communication in the maternofetal interface [57]. Overall, trophoblast debris is a critical component that regulates immune and vascular responses in the placenta, with multiple consequences in maternofetal well-being. 

#### 2.4.2. Endocrine Activity of the Placenta

The endocrine function of the placenta is a major hallmark of the placenta. STBs are the most important source of hormones in the placenta, although it seems that other cell types in the placenta, such as the placental bed giant cells derived from EVTs, may also participate in the endocrine milieu in the placenta [58,59]. The production of several hormones in the placenta is crucial for pregnancy onset and maintenance, with multiple local and systemic effects. Costa [60] summarized some of the most important pregnancy hormones, emphasizing the role of human chorionic gonadotropin (HCG), estrogens, progesterone, placental growth hormone (PGH), placental lactogen, adiponectin, and other adipokines, leptin, resistin, pregnancy-associated plasma protein-A (PAPP-A), activin A, inhibin A, placental protein-13 (PP13), and kisspeptin. All of these hormones are crucial in regulating placentation, immune tolerance, and fetal growth and development in healthy pregnancies. Moreover, altered blood levels of most of these hormones are important biomarkers to study in pregnancy-related complications [61]. 

#### 2.4.3. Placental Barrier

The placenta also represents an important mechanical, chemical, and immunological barrier, exerting diverse actions to protect the fetus. As previously defined, the immunology of the placenta is quite diverse, playing a crucial role in pregnancy homeostasis. Moreover, the immune system and trophoblast also collaborate to reach immune defense against pathogens, including bacteria, viruses, or protozoa, which could be associated with several adverse events [62]. In these times, the severe acute respiratory syndrome coronavirus 2 (SARS-CoV-2) pandemic responsible for the coronavirus disease 19 (COVID-19) has importantly hit the global population, with an important demographic and socioeconomic burden [63]. Pregnant women are considered a group vulnerable to COVID-19 [64]. Prior research has described the central role of the placenta against SARS-CoV-2 infection. The antiviral response of the placenta comprises the presence of Toll-like receptors (TLRs) and RIG-I-like receptors (RLRs) in the trophoblasts, leading to the activation of the nuclear factor-kB (NF-kb) and type III interferon (IRF3) to orchestrate the antiviral response. Moreover, microRNAs from the chromosome19 miRNA cluster (C19MC) are also major mediators of this defense [65]. Immune responses occurring in the placenta are especially effective, as cases with vertical transmission are rare, though they exist, also aiding in softening the cytokine storm in severely ill patients and in mitigating an exacerbated immune response [66]. Moreover, the presence of an own microbiome in the placenta should also be considered in the relationship between the immune system and microorganisms. Unfortunately, evidence confirming that the presence of an own microbiome in the placenta [67] are not consistent, and further studies are required in this field to unravel the presence and/or functions of the microbiota in the placenta. 

The defensive role of the placenta extends far beyond the immune system and infections. Xenobiotics are essentially drugs or outer components that are not naturally produced in the body. Previous studies have found that these xenobiotics may cross the placenta to some extent via passive or active mechanisms [68]. Hopefully, the placenta and, in particular, STB counts with a range of enzymes involved not only in the synthesis of different hormones or metabolic reactions but also in detoxification and efflux of xenobiotics, acting in a similar manner to the hepatocytes in the adult [69]. These enzymes are known as xenobiotic-metabolizing enzymes (XMEs), participating in the biotransformation and elimination of maternal and fetal hormones, dietary compounds, drugs, and environmental chemicals [70]. Among these enzymes, of note is the role of cytochrome P450 (CYP), also found in hepatic cells. For instance, at the early stages, the placenta expresses CYP2C, CYP2D6, and CYP3A7, whereas, at term, CYP4B1 and CYP19 (steroid aromatase) are expressed more often. CYP1A1 is prominently induced by exposure to cigarette smoking [71]. Other enzymes of note are the multidrug resistance protein (MRP) family and P glycoprotein. This family, composed of MRP1, MRP2, and MRP3, is differentially expressed in the apical membrane of STBs and the fetal endothelium and is critical for protecting the fetus from the entry of organic anions [72]. The P glycoprotein encoded in the gene MDR1 limits the exposure of fetal hydrophobic and cationic xenobiotics acting as an active pump that leads xenobiotics back into maternal circulation [73]. If any pathological condition is established, the enzyme 11β-hydroxysteroid dehydrogenase-2 (11β-HSD2) is highly expressed in the placenta in order to transform maternal cortisol into cortisone, preventing damage associated with excessive maternal stress [74]. Of note, similar to the nutrient transfer function, 11β-HSD2 is differentially expressed in male and female fetuses. Stark et al. [75] found that this enzyme exerts reduced activity in newborn males relative to females, which may have important implications in the different male morbidities and mortalities following preterm birth. Likewise, the placenta also defends the fetus from excessive oxidative stress, which could be related to several damages, both for the mother and fetus [76]. Oxidative stress is the result of excessive oxidants or free radical production, mainly represented by reactive oxygen species (ROS) and reactive nitrogen species (RNS). Antioxidants are molecules (vitamins or enzymes) implicated in the defense of oxidative damage derived from ROS and RNS. In non-pathological pregnancies, the placenta expresses a wide variety of antioxidants, therefore preventing the development of oxidative stress, which is associated with adverse pregnancy outcomes [77,78].

#### 2.4.4. Placenta and Maternofetal Programming

As shown, the placenta is an essential link between the mother and fetus. Any changes in the maternal organism may affect the placenta, and this could have noteworthy implications in the fetus. Alterations in the growth, vascularization, nutrient, and waste product exchange, hormone production, or metabolism may have long-term effects on the offspring’s life [79]. Thus, any insult received in utero at a critical developmental stage may lead to fetal programming, which can determine the development of different diseases in adulthood [80]. From an evolutionary perspective, the placenta is the first way by which the fetus makes contact with the outer environment, receiving endogenous and exogenous signals from the mother. Past and recent environments are major determinators of maternal phenotype and, together with fetal genotype, are related to placental functionality. The fetus responds by increasing its Darwinian fitness, ensuring the probability of reproduction, and preparing the fetus to face the real world after birth [81]. Fortunately, environmental exposure throughout the life of the offspring also affects phenotype, although fetal programming should also be considered. For instance, maternal undernutrition in pregnancy negatively impacts placental development and function, also limiting fetal growth and development [82]. This fact may be understood for the fetus as a signal of food scarcity, which may lead to the activation of the “thrifty phenotype”, fetal programming that is associated with increased risk from suffering from type 2 diabetes, metabolic syndrome, and cardiovascular disease in adulthood [83]. Maternal overnutrition may also lead to adverse developmental and long-term outcomes for the offspring due to the activation of multiple epigenetic mechanisms [84]. Recently, Connor et al. [85] compared the effects of undernutrition versus a high-fat diet in placental morphology and functionality. Interestingly, they obtained different adaptative responses of the placenta to both situations, concluding that, compared with undergoing less maturity and inefficient placental transport, overnutrition was associated with variations in multiple placental markers, adapting to the excessive nutrient supply. However, the relevance of ensuring an adequate diet and environment during pregnancy to influence proper development of the placenta and fetus is undeniable. In this sense, different approaches must be considered here, including the introduction of a proper dietary context and an active lifestyle, with the implementation of different and adapted training [86,87]. Limiting exposure to cortisol and stress as well as adequate sleep hygiene will favorably influence the placenta development and functionality, also having positive short and long-term outcomes for the fetus [88,89,90]. 

On the other hand, despite the relevance of placental structure, development and functioning have been extensively researched for fetal programming, compelling evidence also evaluates the pivotal role of this organ for maternal health. During pregnancy, virtually all systems and organs of the women undergo different physiological adaptations, according to the fetal necessities [91]. The cardiovascular system is prominently altered during pregnancy, showing a set of changes that are critical for ensuring an adequate blood supply to the placenta and the fetus [92]. Emerging evidence supports that if the uteroplacental circulation is not well-established, there is an increased risk of short-term and long-term cardiovascular disease for the mother [93]. Besides, it seems that changes in the inflammatory response in the placenta and on its genome and epigenome may drive substantial consequences not only for the fetus but also for maternal health [94,95]. As it will be subsequently discussed, there are different vascular disorders of pregnancy whose pathophysiological basis resides on altered placental perfusion, hence highlighting the importance of this organ for the maternofetal well-being. Moreover, a very recent longitudinal study of 33,336 women followed for 50 years showed that the placental weight to birthweight ratio was associated with long-term maternal mortality [96]. In other words, the status and development of the placenta are also crucial for maternal health during and after gestation, as well as to prevent long-term morbidity and mortality.

Collectively, the placenta exerts multiple functions that are crucial for pregnancy success. Moreover, these activities are crucial for maternal and fetal well-being both in the short and long terms, therefore supporting the capital importance of this organ in a human’s life. In the next section, we summarize some of the most relevant changes occurring in the placenta and the consequences under certain pathological conditions.

## 3. Describing the Placenta in Pathological Conditions

### 3.1. The Role of Placenta in Preeclampsia

#### 3.1.1. Introduction

PE belongs to a set of diseases defined as hypertensive disorders of pregnancy, which also include chronic hypertension, gestational hypertension, and chronic hypertension with superimposed PE. Epidemiological data indicate that PE has a prevalence of 3–5% among all pregnancies, whereas the presence of any hypertensive disorder in pregnancy is estimated to be 10% [97]. PE is a disorder of pregnancy, associated with new-onset hypertension, often accompanied by new-onset proteinuria, although this condition may be presented in the absence of this clinical sign [98]. The main diagnostic criteria of PE consist of the presence of >140 mmHg systolic blood pressure and >90 mmHg diastolic pressure manifested after 20 weeks of gestation. Traditionally, patients with increased blood pressure over 160 mmHg systolic blood pressure and 110 mmHg diastolic blood pressure were diagnosed with severe PE. However, following the current recommendations of the International Society for the Study of Hypertension in Pregnancy (ISSHP), this distinction should not be made anymore, as independent of the blood pressure, PE can deteriorate rapidly and without warning [99]. In the absence of proteinuria, new-onset hypertension may be manifested with thrombocytopenia, renal insufficiency, impaired liver function, pulmonary edema, and new-onset headache unresponsive to medication and not accounted for by alternative diagnoses or visual symptoms [100].

Multiple risk factors for suffering from PE have been identified, including preexisting medical conditions, such as antiphospholipid syndrome, hypertension, or insulin-dependent diabetes; family history or having suffered a prior event of PE; obesity; age (≥40 years old), assisted reproductive techniques; as well as nulliparity or multiple pregnancies [101,102]. From a clinical perspective, two main types of PE exist: (1) early-onset PE (EO-PE), also defined as placental PE, and (2) late-onset preeclampsia (LO-PE). This classification depends on the time of initiation of clinical symptoms, with EO-PE occurring before 34 weeks and LO-PE occurring after 34 weeks [100,103]. Moreover, the impact of PE for the fetus and the mother, serum markers, heritability, and clinical features are quite different for each presentation [104]. 

The consequences of PE, and especially EO-PE for both the mother and fetus, are numerous. For the mother, the most severe and life-threatening issue could be the development of a cerebrovascular hemorrhage, and as previously discussed, an increased risk of suffering from cardiovascular diseases later in life [105]. HELLP syndrome (hemolysis, elevated liver enzymes, and low platelet count) is another presentation occurring in 10–20% of women with PE, sharing many pathophysiological mechanisms [106], although HELLP syndrome has also been reported without occurring PE [107]. Eclampsia can also occur as a result of PE, consisting of the development of generalized tonic-clonic seizures more often occurring antepartum, 20 weeks after gestation, intrapartum, and postpartum, although some exceptional cases have reported the onset of eclampsia before 20 gestational weeks as well [108]. For the fetus, one of the most worrisome consequences is fetal growth restriction (FGR), as will be subsequently discussed [109]. Other possible adverse outcomes include oligohydramnios, increased risk of stillbirth, and in many cases, PE could be related to iatrogenic preterm birth, which may drive to the development of infant respiratory distress syndrome, intraventricular hemorrhage, sepsis, bronchopulmonary dysplasia, and neurodevelopmental disability [105]. Besides, there is also a profound remodeling of the vascular system of the infants affected with EO-PE, especially for preterm-born offspring [110]. 

#### 3.1.2. Preventive and Therapeutic Approaches

The only definite cure for PE and its possible complications is delivery. On the other hand, prior studies have reported some benefits from using low-dose aspirin (LDA) as a prophylactic method in high-risk populations [111]. In more detail, the ASPRE (Combined Multimarker Screening and Randomized Patient Treatment with Aspirin for Evidence-Based Preeclampsia Prevention) trial show that 150 mg per day from 11–14 until 36 weeks of gestation reduced the risk of suffering from PE by 62%, hence supporting the relevance of aspirin as a unique prophylactic agent currently available [112]. The most significant effects occurred when LDA was administered before 16 weeks of pregnancy. Indeed, the initiation of this treatment after this time did not diminish the risk of suffering from PE, according to a meta-analysis conducted by Bujold et al. [113]. Besides, the prophylactic benefits from using LDA were observed to prevent preterm PE but not full-term PE, and only if starting before 16 weeks [114]. Compelling evidence has also found that calcium supplementation before and early in pregnancy may reduce the risk of women experiencing the composite outcome PE and pregnancy loss at any gestational age [115]. This could be especially useful in low-income countries, where calcium deficiency is more common [116]. Other strategies such as physical exercise, rest, reduced salt intake, and other nutritional interventions have shown insufficient evidence to be recommended as preventive measurements for PE [117]. 

Likewise, an early prediction of PE allows for timely initiation of preventive therapy. Moreover, conducting a rapid and early diagnosis of PE is equally important in order to perform continuous observation of the affected patient. In this sense, as it will be subsequently discussed, there are a set of different biophysical and biochemical markers being explored as major clinical features to predict the onset of PE well as to perform an early diagnosis [118,119]. 

Nowadays, the fact that further knowledge on the biological mechanisms of the disease would be of great aid in the clinical management of such a harmful condition cannot be denied. The placenta has a central role in the pathogenesis of PE and, more prominently, in EO-PE. Currently, an accepted hypothesis explains PE as being a two-stage disease involving the following steps: stage 1 (preclinical), characterized by defective spiral artery remodeling and trophoblast invasion, leading to cell ischemia in the placenta, with an imbalance between anti-angiogenic and angiogenic factors in favor of the former. This anti-angiogenic status may be widespread to the endothelium of the different organs, leading to stage 2 (clinical), in which the maternal syndrome is manifested and defined by systemic endothelial dysfunction accompanied by vascular inflammation, oxidative stress, and the disruption of several serum markers [120,121,122]. 

#### 3.1.3. Pathophysiology of Early Onset/Placental Preeclampsia

##### Defective Spiral Artery Remodeling and Trophoblast Invasion

Failures in uterine spiral artery remodeling and trophoblast invasion are the first pathophysiological events involved in the EO-PE and FGR [123]. However, deficient spiral artery remodeling and trophoblastic invasion are difficult to study in humans, as these processes occur in the earliest stages of pregnancy and the samples are available after the first trimester, before presentation of maternal disease, or at term, after disease presentation [124]. Despite having some limitations, animal studies have shed light on the early pathogenesis of PE, especially mice and rat models. Contrary to human disease, PE must be induced in animals surgically, pharmacologically, or genetically. Moreover, the translation of these models may have some important limitations due to the different placental structures. In this context, the ASB4 deletion murine model and the Dahl S rat have been widely studied to unravel the mechanisms involved in impaired spiral artery remodeling and trophoblast invasion [125]. The precise cause of the defective spiral artery remodeling and trophoblast behavior remains elusive. The hypothesis is that failures in the trophoblast lineage at any stage of early development affect the differentiation of villous trophoblast, which ultimately is responsible for the pathogenesis of PE [56]. The origin of this aberrant functioning is unknown, although different factors have been proposed here, including genetic causes, intrinsic placental alterations affecting the trophoblast and immune system behavior, as well as extrinsic or maternal determinants [121]. All of these factors may lead to the development of acute atherosis in the spiral arteries, which appears in between 20 and 40% of women with PE [126]. In turn, this could be one cause of placental infarction that can be documented in 70% of patients with severe PE and 40% of mild PE [127]. 

##### The Antiangiogenic Status

Persistent placental ischemia/hypoxia derived from aberrant spiral artery remodeling and trophoblast invasion is another critical event involved in the pathogenesis of PE [128,129]. Sustained and augmented levels of hypoxia-inducible factor (HIF-1α) are associated with the enhanced production of anti-angiogenic components, such as soluble fms-like tyrosine kinase-1 (sFlt-1) and soluble endoglin (sEng), accompanied by reductions in pro-angiogenic markers such as VEGF and placental growth factor (PlGF) [130]. sFlt-1 is the soluble form of the Flt-1 receptor, also designated as VEGF receptor-1. Likewise, sEng is the soluble form of the Eng receptor, and in broad terms, both Flt-1 and Eng receptors are essential mediators of the angiogenesis process in tissues mainly due to their interaction with VEGF and PlGF [131,132]. Under hypoxic conditions, the preeclamptic placenta shows a decrease in PlGF production together with an increase in the expression of both Eng and Flt-1 receptors as well as their soluble forms released into maternal blood [133]. sFlt-1 binds to free VEGF and PlGF, thereby limiting the bioavailability of these angiogenic components [134]. Chronic hypoxic trophoblasts are responsible for a sustained sFlt-1 release that indeed is sufficient to cause endothelial dysfunction in vitro [135]. This effect is amplified when sFlt-1 acts with sEng, which impairs the binding of TGF-β1 to its receptors and its downstream signaling in the vasculature [136]. The binding of sFlt-1 to free circulating VEGF and sEng to TGF-β1, together with a decreased PlGF production by the trophoblasts, are at some extent responsible for the impaired local angiogenesis in the placenta and the systemic endothelial dysfunction related to PE. Additional evidence supporting the central role of these factors in the pathogenesis of PE is their relevant use in clinical practice as central biomarkers. For instance, measuring the sFlt-1/PlGF ratio allows us to define the clinical diagnosis of PE [137,138]. A PROGNOSIS (Prediction of Short-Term Outcome in Pregnant Women with Suspected Preeclampsia Study) study showed that an sFlt-1/PlGF ratio of 38 or lower had a negative predictive value of 99.3%, whereas the positive predictive value of an sFlt-1/PlGF ratio of 38 or above was 36.7% within 4 weeks. This means that a low ratio of sFlt-1/PlGF (38 or lower) can be used to predict the short-term absence of PE in women in whom the syndrome is suspected clinically [138], although a higher ratio did not have to be related to the presence of PE. Hence, analyzing this ratio could also serve to prevent inappropriate hospitalization, which has a significant economic impact [139]. Therefore, cumulative evidence supports the clinical use of measuring both sFlt-1 and PlGF in women with PE. 

##### Vascular Inflammation

Previous studies have identified a central role of innate and adaptative immune cells in PE. dNKs and decidual macrophages are hyperactivated in PE in response to sustained hypoxia and excessive trophoblast cell debris or trophoblast necrosis [140,141]. For instance, enhanced production of angiotensin II type 1 receptor autoantibodies (AT1AA) is observed in patients with EO-PE, which is a major consequence of a renin–angiotensin–aldosterone system (RAAS) dysfunction [142]. Some authors have hypothesized that the abnormal placental development causes an exacerbated release of RAAS components and other molecules implicated in the regulation of this system, such as micro RNAs or AT1AA, probably in exosomes [143]. AT1AA is an agonist antibody secreted by B cells responsible for the activation of the angiotensin II type 1 receptor, similar to angiotensin II, with multiple consequences. Among the main targets of AT1AA, the activation of the MAP kinase (MAPK) pathway tissue factor, plasminogen activator inhibitor-1 (PAI-1), an impaired trophoblast invasion, oxidative stress markers, and augmented production of sEng through TNFα induction should be highlighted [144]. AT1AA overproduction is not the only mechanism by which the immune system mediates its pathophysiological role in PE. Simultaneously, a differentiation of Th cells into Th1 and Th17 with diminished Treg activity also influences the abnormal inflammatory response found in PE [145]. These changes are accompanied by substantial changes in the cytokine profiling, with an overproduction of type I (pro-inflammatory) cytokines IL-6 and TNF-α and a decrease in type II (anti-inflammatory) IL-4 and IL-10 [146]. In this context, some authors claimed that endometrial mesenchymal stem cells are relevant in the immune system, mediating immunosuppressive effects in the local tissue and allowing for trophoblast invasion in normal pregnancies. The precise role of these cells in the etiopathogenesis of PE remains unknown [147], although some in vitro studies have reported some promising results from using this type of cell to reduce the Th1 pro-inflammatory differentiation [148]. Further research in this field may aid in the immunoregulation occurring in the placental environment at the early stages. Some authors hypothesized that an altered placental microbiome might shed inflammatory molecules, such as lipopolysaccharides (LPS), with a resultant inflammatory switch that accompanies abnormal placentation and maternal endothelial cell activation [149]. The excessive immune activation contributes to endothelial dysfunction via several mechanisms, including the release of endothelin 1 (ET-1), ROS, and an increase in vasoconstrictors such as the proper AT1AA and angiotensin II along with a decrease in vasodilators such as nitric oxide (NO) and prostacyclin [150]. ET-1 is a direct consequence of endothelial dysfunction, related to a powerful vasoconstrictor response. ET-1 is produced due to VEGF inactivation or inhibition. Then, ET-1 triggers the production of ROS and oxidative stress in the placenta, which responds with enhanced production and release of sFlt-1, creating a vicious cycle [151].

##### Oxidative Stress

Oxidative stress is widely accepted as a major determinant of PE and placental diseases. Contrary to normal pregnancies, PE is characterized by increased ROS and oxidative molecules, with a decrease in antioxidant systems [152]. Virtually all placental cell types are sources of oxidative stress, including trophoblasts, endothelial cells in the placenta, Hofbauer macrophages, or stromal cells in the villi [153]. The sustained hypoxia leads to excessive inflammation and oxidative stress, two closely related conditions [154,155]. Furthermore, excessive oxidative stress may lead to a disruption in different placental cells, affecting some critical events, such as apoptosis or autophagy, participating in the pathogenesis of PE [156]. Among the most important consequences of oxidative stress, a decrease in the placental endothelial nitric oxide synthase (eNOS) strongly diminishes NO production via several mechanisms [157]. In this sense, some studies have found an inverse correlation between NO levels and levels of sFlt-1 and sEng in women with PE, thereby suggesting that somehow anti-angiogenic factors may inhibit the production of NO [158]. The diminished NO production is also potentially related to the reduced bioavailability of its precursor, L-arginine, both being observed in women with PE [159]. These alterations may promote changes in the renal and cardiac microvasculature as well as a reduction in the number of fetal nephrons in vivo, which can possibly also occur in humans [160]. Notwithstanding, a clear implication of oxidative stress is seen in the etiopathogenesis of PE: the use of antioxidants such as vitamin C, E, or n-acetylcysteine has failed to show any clinical benefit [161].

##### Endocrine Disruption

Many of the hormones produced by the placenta may be partly responsible for the onset and development of PE. For instance, reduced levels of hCG are secreted by STBs in the first trimester, but an increased detection in the second trimester appears to be associated with a later diagnosis of PE [162]. These differences were due to the pro-angiogenic activity of hCG, and the higher presence of this protein seems to be a compensatory mechanism of the placenta to the sustained ischemia. Moreover, decreased levels of hyperglycosylated Hcg (HhCG) secreted by EVT in the second trimester was also associated with a higher risk of PE. Importantly, HhCG, but not hCG, is involved in deficient trophoblast invasion, which may support its possible pathophysiological role in PE [163]. Other altered hormones associated with the pathophysiology of PE include androgens (testosterone and androstenedione) and leptin, which in turn are correlated with estrogen levels [164]. Other hormones not produced by the placenta, such as cortisol, arginine vasopressin (AVP), epinephrine, norepinephrine, natriuretic atrial peptide, brain natriuretic peptide, and melatonin also appear to be implicated in the pathogenesis of PE [165], hence concluding the complex role of multiple hormonal systems in these pregnancies. 

##### Placental Aging and Damage

As mentioned above, increased cell death is observed in the placental tissue of women with EO-PE. Apart from oxidative stress, the enhanced apoptosis observed in the placental tissue related to PE is also attributable to the triggered inflammation and chronic hypoxia. Whereas in physiological pregnancies, the apoptosis process is exclusively regulated by the immune system through the extrinsic pathway in an FAS ligand (FASL) manner, the pathologic environment in PE leads to the activation of the intrinsic route and a decrease in anti-apoptotic proteins. This concludes with enhanced apoptosis of EVTs and STBs, with increased syncytial knots, a marker of accelerated aging in the placental tissue and in EO-PE [166,167]. The premature aging of placental tissue is also supported by the dysregulation of several hallmarks of aging, including telomere shortening, cell senescence, loss of proteostasis, epigenetic variations, and mitochondrial dysfunction, among others [168,169,170,171]. The acceleration in the aging process of the placenta may be caused by the pathological environment in this organ while contributing to an exacerbation of the disease. 

**Figure 2:**
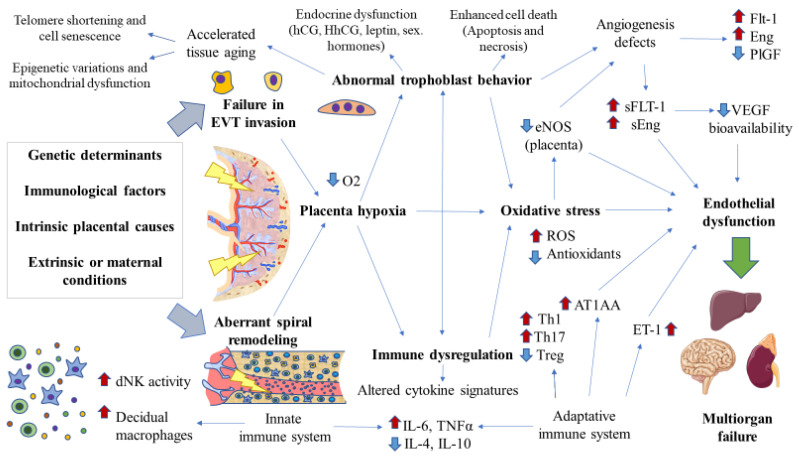
An overview of the main mechanisms involved in the pathogenesis of early-onset preeclampsia. As described before, aberrant spiral artery remodeling and failures in trophoblast invasion trigger placental hypoxia, leading to an exacerbated immune response affecting the innate and adaptative immune systems and cytokine production. In turn, an abnormal trophoblast behavior is also observed in the placenta, including accelerated aging, endocrine dysfunction, enhanced cell death, and angiogenesis defects, emphasizing the relevance of an imbalance between anti-angiogenic (sflt-1 and sEng) and pro-angiogenic factors (VEGF and PlGF). Almost every cell type in the placenta increases ROS production and decreases antioxidants, which are associated with oxidative stress, affecting some crucial components in the placenta, such as endothelial nitric oxide synthase, with detrimental effects on angiogenesis. Overall, oxidative stress, trophoblast alterations, and the inflammatory environment lead to systemic and profound endothelial dysfunction, eventually affecting different organs in the body and leading to the onset of clinical manifestations.

In this section, we summarized the multiple alterations occurring in the placenta tissue both in the preclinical and clinical stages of PE, with substantial consequences not only for this tissue but also for the mother and fetus. The main results summarized in this section are represented in Figure 2. Further insights into the role of the placenta in EO-PE should significantly help deepen our understanding of the origins and development of such an intricate disease, also identifying novel biomarkers with prognostic or predictive value, or perhaps prophylactic approaches. 

#### 3.1.4. Pathophysiology of Late-Onset Preeclampsia

Contrary to EO-PE, designated as placental PE, LO-PE pathogenesis is often related to maternal pathophysiology, and neither the placenta nor fetus suffer the same significant effects as in EO-PE [172]. LO-PE presents higher rates of incidence than EO-PE, and different risk factors have been identified for both conditions. Furthermore, EO-PE is associated with a higher incidence of fetal death and maternal risk of many cardiovascular, respiratory, nervous, hepatic renal, and other morbidities in comparison with LO-PE [173,174]. These differences may be attributed to the fact that, in LO-PE, the spiral arteries seem to maintain normal behavior, with no evidence of an aberrant trophoblast invasion. The etiology of this condition is speculated to probably be more ligated not to placental but to maternal extrinsic factors [56,174]. In this sense, some authors proposed that both EO-PE and LO-PE are consequences of STB stress [175]. However, in contraposition to EO-PE, LO-PE may be secondary to intraplacental (intervillous) malperfusions due to mechanical restrictions as no evidence of altered spiral artery remodeling exists. In this line, a growing amount of evidence endorses the implication of the cardiovascular system in the origin of LO-PE, resulting from the inability of the maternal heart to meet the increased metabolic demands of an overgrown fetoplacental unit [176]. Indeed, an abnormal Doppler assessment may be reported not only in uterine arteries but also in other unrelated blood vessels [177]. Thus, the main differences between both conditions in pathophysiological terms may be the duration and causes of dysfunctional uteroplacental perfusion, although the response and behavior of the placenta and stage 2 are quite similar [175]. Moreover, notwithstanding, more studies are required to compare EO-PE with LO-PE biomarkers, and some differences have been found between them, including oxidative stress (superoxide production is higher in EO-PE in comparison with LO-PE), angiogenesis (further decrease in tyrosine kinase endothelial receptor (Tie-2) and increase in Flt-1 receptor in EO-PE), inflammatory markers (upregulation of the intercellular adhesion molecule 1 (ICAM) is more marked in EO-PE), and hormone functionality (PP13 is more decreased in EO-PE) [104]. Additionally, the placenta of women with EO-PE shows further hypomethylation of different circadian clock elements in comparison with LO-PE and non-pathological placentas, indicating a different epigenetic pattern in these genes [178]. The possible differential role of other pathological markers was previously described in EO-PE versus LO-PE, although the results obtained are not consistent [104]. Collectively, these studies revealed noteworthy variations in the placental behavior in EO-PE versus LO-PE. The fact that EO-PE is established at the early stages of placentation, leading to prolonged hypoxia, inflammation, or oxidative damage, may explain these differences. Nevertheless, LO-PE may be taken cautiously as some of the pathophysiological mechanisms reported in EO-PE also exist in this condition. 

### 3.2. Placenta in Fetal Growth Restriction

#### 3.2.1. Introduction

FGR describes a condition characterized by an abnormal umbilical artery Doppler and is frequently related to a fetus small for gestational age (SGA) (less than the tenth percentile of weight at birth) [179,180,181]. However, it is important to make a distinction between FGR and SGA, as recent works recognize that both SGA and FGR may be considered separately. For instance, infants with SGA could not be growth restricted, and newborns with birth weights > 10th percentile can be growth restricted. Thus, the agreed-upon definition of FGR includes birth weight less than the third percentile or three out of the following criteria: birth weight < 10th percentile, head circumference < 10th percentile, length < 10th percentile, prenatal diagnosis of FGR, and the presence of any pregnancy complication [182]. Other variables such as placental, brain, or liver weight lower than 10th percentile; brain-weight-to-liver-weight ratio higher than 4; placental-weight-to-birth-weight ratio higher than 90th percentile; and histologic or gross features of placental insufficiency/malperfusion may also be used to identify fatal cases of FGR [183]. In the case of SGA infants, it could be categorized into two major groups: (A) Constitutionally normal infants with birth weight less than the 10th percentile due to inherent factors like maternal height, weight, ethnicity, and parity, and (B) SGA infants secondary to FGR [181].

FGR affects 10–15% of pregnant women and is the second most frequent cause of perinatal mortality and morbidity [184]. The percentage of FGR births is different across regions, with the highest-burden being in Asia, accounting for nearly 75% of all global cases of FGR [185]. It is of great importance to understand that this does not mean that in these regions, 3/4 births are affected with FGR. Conversely, due to the limitation of available data in other regions, the total fertility rates, the number of inhabitants in Asian countries like India or China, and more prominently because of the relationship of some of these countries with the different risk factors that will be subsequently discussed, more cases have been reported in Asia in comparison to other continents.

The origin of the disease remains elusive. However, it is thought to be caused by an interaction of environmental and genetic factors with either a fetal, placental, or maternal origin [186]. 

The maternal factors involved in FGR are various. One of them is, as previously stated, a PE diagnosis. Prevalent undernutrition in women from developing countries is key to the origins of FGR and consequent stunting, wasting, and other predisposing factors for child mortality [187]. The opposite case, overnutrition due to abnormal maternal diet and pregnancies associated with obesity or diabetes, is also relevant due to over-insulinisation and derived vasculopathies [188,189], as well as advanced maternal age [190]. Other factors that increase the risk of FGR are toxic substances consumed by smoking, alcohol consumption, and poly drug use, which imply lower food consumption and, thus, lack of fetal weight gain [191,192,193]. Not less important is the exposure to environmental chemicals, such as bisphenol A and phthalates, which impair the functions of placental hormones such as gonadotropin, estrogens, progesterone, prolactin, or growth hormone, especially in the first half of pregnancy [194,195]. Avoiding contact with polycyclic aromatic hydrocarbons is also important, especially in the first trimester of pregnancy, when they cause more severe adverse effects on fetal growth [196]. Some cases in which diffuse chronic villitis of unknown etiology can also be associated with FGR [179]. Additional maternal factors include maternal hypertension, exposure to stressful conditions, genetics, or high altitudes [197]. Fetal causes encompass some congenital malformations like congenital heart disease, congenital diaphragmatic hernia, or trisomies 13, 18, and 21 [198]. Infections caused by HCMV, rubella, or *Plasmodium falciparum*, and multiple pregnancies are also common causes of FGR [179,199]. The placental causes of FGR are mainly related to altered nutrient transport in the placenta and, more specifically, with amino acids, glucose, fatty acids, and oxygen intake [200]. Other placental factors include an incorrect umbilical cord insertion, placental tumor, a single umbilical artery, and circumvallate placenta [197].

Despite the different origins that this condition may entail, it must be remarked that FGR is, in fact, a consequence of placental vascular pathology, concretely a chronic insufficiency of oxygen and nutrients for the fetus due to impairments in maternofetal circulation [201]. The accompanying problems that newborns may have include perinatal asphyxia, hypothermia, hypoglycemia, and polycythemia, and later, they may also face growth retardation and neurodevelopmental problems, with all of these echoed in their health in adulthood [202]. Consequences in adulthood include, above all, non-communicable diseases such as metabolic syndrome, renal and cardiovascular disease, and neuro-inflammation [195]. Adaptative changes of FGR persist after birth. Although neonates show smaller left ventricular dimensions, either in symmetric or asymmetric FGR, systolic function seems less altered when compared with non-FGR neonates [203,204]. Other studies find an apparent dysfunction of systolic and diastolic signs, and coronary flow is significantly increased in neonates with FGR, but continuous monitoring of extrauterine life shows a similar heart rate in comparison to infants without FGR [205,206]. Despite these results, further research is required to denote how extra effort by a premature neonate for remodeling cardiac dimensions and functions—as it could not take place in placenta—may have its short and long-term sequelae echoed in the development of cardiovascular diseases in adulthood [207]. In contrast, embryological alveolar formation is key; uncompleted lung function configurations have shown more difficulties; and epidemiological data show correlations between FGR, low birth weight (LBW), and respiratory morbidity from infancy progress with age to adulthood [208]. High concentrations of surfactant protein D in the umbilical cord blood of premature fetuses with FGR are determinants of asthma and chronic obstructive pulmonary disease in later life [209]. Abnormalities in the RAAS promote fetal renal tubular maldevelopment [210]. 

#### 3.2.2. Pathophysiological Role of the Placenta

As mentioned above, FGR may appear as a consequence of PE, especially EO-PE, sharing common pathophysiological mechanisms in the placenta. For instance, a subjacent deficient remodeling of uterine spiral arteries can be presented in FGR, just as in the case of EO-PE [211]. Malperfusion causes the privation of nutrients and oxygen to support normal aerobic growth of the fetus and then, cell stress, selective suppression of protein synthesis, and hence poorer cell proliferation with a consequent reduced villous volume—lower surface area for maternofetal traffic of substances—and villous damage [212]. The subjacent alterations in intrauterine hydrostatic pressure gradients denote that these disturbances in maternofetal circulation alter metabolism and placental immune function. Morphological and metabolic changes are more severe in cases of FGR associated with PE [211]. New magnetic resonance imaging technologies today allow for watching the redistribution of circulation and incompetent oxygen and substrate transport in response to hypoxia in FGR, translating these into slower fetal brain growth and fetal growth in general [213]. 

Most severe cases determine infarction and fibrin deposition. Massive intervillous fibrin deposition, so-called maternal floor infarction, compromises villous tree. It constitutes a placental disorder in which fibrinoid material is deposited within perivilli and intravilli, correlated with hypovascular/avascular villi and elevated plasma cell deciduitis, with inflammatory infiltration being determinant for villous architecture lesions [214,215]. 

The placenta is a pivotal structure responsible for the pathophysiology of FGR, and exposure to multiple environments during pregnancy may lead to detrimental effects in this organ. For instance, alcohol consumption and its metabolic products entail multiple changes in the placenta, including oxidation of lipids and proteins, DNA damage, mitochondrial dysfunction, apoptosis, and cellular injury [216]. This leads to a prominent placental dysfunction, decreased placental size, endocrine changes, impaired blood flow, and nutrient transport, increased rates of stillbirth and abruption, umbilical cord vasoconstriction, and LBW [217]. In this context, recent studies have demonstrated the association between alcohol consumption with impaired insulin-like growth factor 1 (IGF-1) signaling. IGF-1 has a pivotal role in mediating cell proliferation, migration, and differentiation, as well as in placentation, thereby showing that the inhibition of this molecular pathway by ethanol may be partly responsible for FGR [216]. Some questions to solve in future studies include what molecular mechanisms underlie ethanol-mediated placental dysfunction and what are the possible adaptative responses of this organ according to the fetal sex [218].

#### 3.2.3. Screening, Predictive, and Diagnostic Biomarkers

Due to the huge impact of FGR, effective screening of this condition is of significance in limiting the detrimental effects of this condition. In this sense, some studies have proven that a combined analysis of fetal biometry and fetal growth velocity is essential for identifying a subset of SGA fetuses that are at increased risk of neonatal morbidity, roughly tripling the detection of SGA infants [219]. 

The simultaneous assessment of maternal and fetal biomolecular markers has been determined to study this complication, with most of them having been mentioned above for PE, sharing the upregulation of anti-angiogenic factors. The measurement of maternal serum PAPP-A is downregulated in thick placenta (>4 cm or >50% of placental length) (evaluated with ultrasound technique) [220] and is associated with poor pregnancy outcomes [221]. Altered serum levels of PAPP-A have been observed to be associated not only with FGR but also gestational diabetes and PE [222,223]. Long-term studies have found that this measurement is also associated with short stature in offspring and maternal diabetes mellitus in later life [224]. For this reason, this biomarker is key in the first trimester for a sooner prediction of placental health and prenatal outcomes to classify patients with increased risk of FGR [225]. Evidence shows that it is also relevant for monitoring the mother and her offspring in later life. In fact, PAPP-A has proven to be reliable as a single predictor for prenatal screening, and when combined with other maternal serum markers, the sensitivity of diagnosis rates increases [226].

Other biomarkers have been used lately to study the impact in newborns with FGR. The role of the extracellular matrix glycoprotein reelin in perinatal neurodevelopment has shown elevated levels significantly associated with cerebral blood redistribution [227]. Angiogenic molecules such as PlGF, which was implicated in PE, are also applicable to FGR. Very low levels of this marker correlate with significantly lower gestational age at delivery and is an indicator of urgent delivery in pregnancies at risk of adverse outcomes [228]. Additionally, according to some prospective studies, calculating the sFlt-1/PlGF ratio can predict PE and FGR after the 34th week [229]. Several prospective studies have emphasized the importance of accomplishing repeated measurements of serum values in pregnant women, such as the sFlt-1/PlGF ratio, as PE and FGR can account for the later stages [230]. Conversely, the plasma levels of sFlt1 and sEng do not show significantly different results for patients with PE compared with patients with FGR [231]. Additionally, the abovementioned anabolic hormones IGF-1 and its binding proteins (IGFBPs) present low circulating levels in FGR, endangering oxygen and nutrient transport across the placenta and, hence, its dysfunction. As IGF-1 plays a key role in fetal growth, several animal models have proposed that the administration of low doses of this hormone restores its function in FGR pathophysiology [184]. Likewise, certain studies found that, from the first trimester, low maternal-free β-hCG concentrations are associated with adverse outcomes, such as FGR, preterm birth, and LBW. On the other hand, abnormally high concentrations in the second trimester were associated with spontaneous abortion and FGR as well [232]. Additionally, recent studies have shown that circulating Epidermal Growth Factor-Like domain 7 (EGFL7) allows us to discriminate between isolated FGR and PE at different gestational ages. Levels are high in both pathological conditions, but the cutoff is significantly different, being much higher in PE [233]. In any case, standardizing screening methods employing maternal serum biomarkers to predict the window of vulnerability and sooner episodes of FGR development are thus necessary.

Finally, the diagnosis and management of FGR rely on the use of ultrasound and Doppler technologies—especially the following parameter: uterine artery Doppler velocimetry (UADV) pulsatility index (PI). Optimized imaging studies are needed for earlier detection and for preventing the late onset of FGR [197] and suppose a more cost-effective multi-parametric testing [234]. Moreover, each case should be addressed individually to determine the appropriate timing of delivery. PlGF from maternal blood can reveal urgent delivery before the 35th gestational week and has been proven to have more sensitivity when fetal flow Doppler ultrasonography cannot identify late adverse events [235]. More efforts are being put into improving monitoring tools with the aim to predict adverse outcomes earlier and to evaluate time to delivery [236]. In the neonatal period, intensive care therapies are regarded as critical research areas [201].

#### 3.2.4. Preventive and Therapeutic Approaches

Once the FGR problem is established, it seems irreversible. Preventing undernourishment in developing countries or sedentary and chronic illnesses in developed countries constitutes a public health concern. 

Nutritional intervention programs are also vitally important in children who were born with FGR and, hence, born with more susceptibility to developing metabolic disorders such as obesity. An example of fetal programming is the IGF-1 gene: from birth, high IGF-1 DNA methylation can be observed to screen infants with higher risks of developing cardiovascular disease or diabetes in adulthood [237]. The growing interest in interventions addressing lifestyle factors has shown evidence for targeting the epigenome, which could prevent morbidities in adulthood. The focus on monitoring individuals who suffered prenatal adversities such as FGR still requires further deepening. Currently, research works about this concern are few, and the guidelines maintain most of the recommendations related to micronutrient supplementation. Supplemented calcium is known to protect against LBW, and magnesium was associated with a reduced risk of SGA [238]. The controlled energy achieved by a balanced diet should always be considered. It would also be interesting to accomplish a study on the effects of zinc, group B vitamins, and other micronutrients in pregnancies with FGR.

In the same context, physical exercise, as one of the pillars of health, should always be individually advised—with adapted intensity for each woman—but the truth is that, for a long time, perceptions about physical activity in pregnancy have been inaccurate [239]. Some rodent models showed a reduced risk of metabolic diseases following FGR thanks to exercise-induced metabolic reprogramming, with improved β-cell functions [240]. Evidence in human studies suggests that some pro-angiogenic factors such as VEGF can be upregulated by exercise during pregnancy and do not correlate with preterm labor or FGR [241], with the gestational weight gain control being promoted instead [242]. Safe physical exercise for pregnant women improves insulin sensitization and glucose tolerance [243]. Likewise, regular workouts before pregnancy ensure better reprogramming of the vascular network, which may aid in preventing FGR.

Moreover, the increased risk of preterm delivery associated with alcohol consumption also supports abstinence and smoking cessation during pregnancy. 

Following the actual recommendations of the FIGO (International Federation of Gynecology and Obstetrics) [244], no medical interventions to prevent FGR have been clearly established. Indeed, cumulative evidence only supports a small benefit from the use of aspirin at low doses to reduce SGA in women at risk, whereas low-molecular-weight heparin shows some promising but still inconsistent results in the prevention of FGR. The only management option in cases with high risks of hypoxia, acidosis, and intrauterine death is iatrogenic preterm birth, with the use of peripartum maternal administration of magnesium sulfate for neuroprotection and corticosteroids for fetal lung maturity in order to prevent adverse neonatal outcomes [245]. Congenital infections, such as those caused by HCMV, may be related to impaired placental development with a high probability of fetal transmission (30–40%), which has permanent hostile consequences in newborns, including not only FGR but also neurological, hearing, and vision defects [246]. Avoiding fetal infections is difficult, although the search for a vaccine to prevent these adversities is still being developed [247].

**Figure 3:**
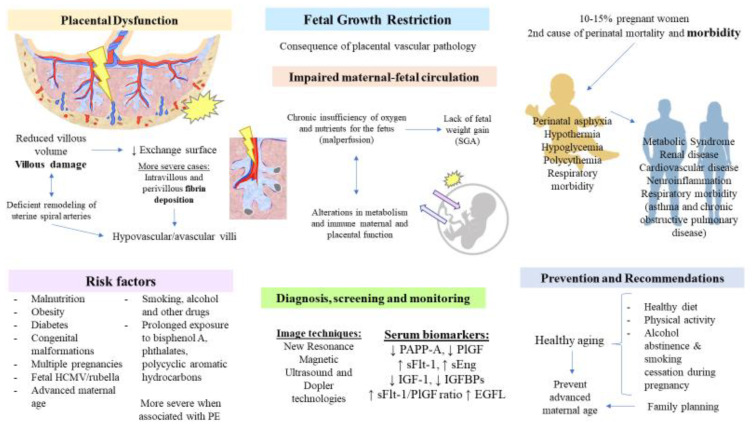
The vital role of the placenta in fetal growth restriction, risk factors, and consequences of the impaired maternofetal circulation. Main diagnosis, screening, and monitoring methods, as well as some preventive measures and recommendations, are also described.

Another factor to bear in mind for pregnancy is advanced maternal age, with advanced age being more susceptible to developing FGR and to experiencing stillbirth. In any case, family planning and social progress may help women find a balance between conceiving children at an optimal biological age and their career/working life in order to avoid disease for themselves and their offspring. The main ideas reviewed in this section are collected in Figure 3.

### 3.3. Chronic Venous Disease, Clinical Manifestations, and Repercussions

CVeD is a multifactorial vascular disorder characterized by an impairment in the venous return, mainly from the lower limbs. Varicose veins (VVs) are the main presentation of CVeD [248], although a broad spectrum of clinical manifestations is classified according to the Clinical-Etiology-Anatomy-Pathophysiology (CEAP) criteria [249]. The term chronic venous insufficiency (CVI) refers to the most advanced stages of CVeD and may include dermatologic alterations and active ulcerations [250]. Notably, CVeD and, more prominently, CVI entail important socioeconomic burdens, with important consequences on the patient´s quality of life [251]. The signature of CVeD is an increase in venous pressure, referred to as venous hypertension. Venous hypertension appears as a consequence of both genetic and environmental factors. The pathophysiology of CVeD involves a profound remodeling and injury of the venous wall, valve incompetence, microcirculatory changes, hypoxia, and a significant inflammatory response secondary to venous hypertension [252]. Women, especially those who are pregnant, represent a vulnerable population that suffers from CVeD [253]. Due to the differential hemodynamic activities occurring in the cardiovascular system, exposure to different hormonal factors, and mechanical action due to proper growth of the fetus, approximately 40% of women undergo CVeD during pregnancy [252]. Moreover, the number of pregnancies also seems to be a major risk factor for suffering from CVeD [254]. Considering that CVeD has not only local but also global consequences is important. Along this line, multiple studies have confirmed the dysregulation of several systemic components, including pro-inflammatory cytokines, epigenetic markers, oxidative stress products, and some circulating parameters (estradiol, homocysteine, VEGF) [255,256,257,258].

The relationship between CVeD and the placenta is notably complex. Prior research suggests that the placenta could represent an important point of study either as a causative agent or as a consequence of CVeD. Indeed, we have demonstrated several changes in the placenta of women who develop CVeD for the first time during the third trimester of pregnancy, with no evidence or diagnosis of any health concern. For instance, as detailed above, the placenta is a central source of hormones in a pregnant woman, and previous studies have noticed the association of some of these hormones and their receptors with the development of CVeD [259,260,261,262]. Moreover, women with CVeD seem to present a higher presence of oxidative stress markers. Particularly, increased levels of NADPH oxidase 1 (NOX-1), 2 (NOX-2), inducible nitric oxide synthase (iNOS), poly (ADP-ribose) polymerase (PARP), and ERK were observed in the placenta of affected women [263]. Moreover, we evidenced augmented malondialdehyde (MDA) levels in the serum of mothers with CVeD and a decrease in the fetus serum pH. Thus, the placenta could be a major source of oxidative stress, although establishing if this may favor the onset and progression of CVeD or whether it is a response is difficult. Furthermore, we also observed evidence of hypoxic damage in the placental villi related to CVeD [264]. The hypoxic environment observed in these placentas was related to an increased number of villi together with enhanced apoptosis and syncytial knot detection in comparison with the women in the control group. Moreover, similar results were also found in the umbilical cord of these women [265], therefore evidencing the association between CVeD with hypoxia and oxidative stress in the maternofetal structures during pregnancy. The various alterations related to CVeD promote an abnormal environment in the placental tissue, leading to the development of different adaptative responses. The sustained hypoxia leads to dysregulation of the local angiogenesis and lymphangiogenesis observed by marked alterations of Flt-1, VEGF, PlGF, cluster differentiation 31 (CD31), and podoplanin (D2–40), which in turn were related to an augmented villous calcification [266,267]. Moreover, a pro-inflammatory environment was also described in the placenta of women with CVeD, accompanied by substantial changes related to the phosphatidylinositol (PI3K) pathway, Wnt/βcatenin, MAPK, the PAPP-A/IGF-1/stanniocalcin-2 axis, and insuline receptor substrate 4 (IRS-4) [263,268]. All of these molecular variations may also be related to a switch in lipidomic profiling in the placenta of women with CVeD [269]. Finally, we observed that profound remodeling affects the placental tissue, with dysregulation of collagen and elastic fibers, including an increase in type III collagen, matrix metalloprotease 9 (MMP-9), and elastogenic processes with a decrease in EGFL7 expression [270,271]. Currently, the impact of CVeD in maternal and fetal well-being is starting to be elucidated. In this sense, we found an important association between VVs in the lower extremity and intrapartum fetal compromise [272]. 

Despite our understanding of the effects and relationship between CVeD, placental composition and function are still in their infancy, and compelling evidence is starting to unravel the harmful effects of this condition in maternofetal structures. This disease likely presents a unique pathophysiological signature in the placenta of pregnant women. Future studies are needed to shed some light on the changes that occur in this organ and the possible implications of this dysregulation. 

## 4. Conclusions

The placenta acts as a pivotal director in fetal and maternal processes during pregnancy and is also critical for understanding healthy and pathological pregnancies. PE, especially EO-PE, is a disease initiated and triggered in the placenta, entailing a set of consequences for both the mother and the fetus, such as FGR. Different factors may be associated with the development of PE and FGR, and some of the different dysregulated products of the placenta may be used in the screening, diagnosis, and monitoring of this condition. Different strategies that focus on correcting placental function are being investigated. Finally, although less established, pregnancy-related CVeD is a condition commonly found in pregnant women, and prior studies have started to demonstrate noteworthy placental alterations in these patients. Further studies are needed to clarify the precise relationship between CVeD and the placenta. 

## Data Availability

Not applicable.
